# Prevention of acute exacerbation of chronic obstructive pulmonary disease after bronchoscopic lung volume reduction with endobronchial valves

**DOI:** 10.1111/crj.13450

**Published:** 2021-10-14

**Authors:** David Abia‐Trujillo, Alejandra Yu Lee‐Mateus, Juan C. Garcia‐Saucedo, Omran Saifi, Neal M. Patel, Felix J. F. Herth, John R. Woytanowski, Ihab Alshelli, Sajive Alevas, Juan P. Uribe Becerra, Adnan Majid, Eric S. Edell, Megan M. Dulohery‐Scrodin, Janani S. Reisenauer, Hiren J. Mehta, Michael A. Jantz, Hawazin K. Abbas, Sebastian Fernandez‐Bussy

**Affiliations:** ^1^ Division of Pulmonary, Allergy, and Sleep Medicine Mayo Clinic Florida Jacksonville Florida USA; ^2^ Department of Internal Medicine Morristown Medical Center Morristown New Jersey USA; ^3^ Department of Radiation Oncology Mayo Clinic Florida Jacksonville Florida USA; ^4^ Department of Pneumology and Critical Care Medicine, Thoraxklinik University of Heidelberg Heidelberg Germany; ^5^ Respiratory Institute Cleveland Clinic Florida Weston Florida USA; ^6^ Division of Thoracic Surgery and Interventional Pulmonology, Beth Israel Deaconess Medical Center Harvard Medical School Boston Massachusetts USA; ^7^ Division of Pulmonary and Critical Care Medicine Mayo Clinic Rochester Minnesota USA; ^8^ Department of Thoracic Surgery Mayo Clinic Rochester Minnesota USA; ^9^ Division of Pulmonary, Critical Care, and Sleep Medicine, College of Medicine University of Florida Gainesville Florida USA

**Keywords:** bronchoscopic lung volume reduction, chronic obstructive pulmonary disease, disease exacerbation, prophylaxis

## Abstract

**Introduction:**

Bronchoscopic lung volume reduction (BLVR) with endobronchial valves (EBVs) has emerged as an important treatment method for patients with severe chronic obstructive pulmonary disease (COPD). Acute exacerbations of COPD (AECOPD) are a frequent complication following BLVR with EBV. However, there is no consensus on the prevention of AECOPD.

**Objectives:**

Our study aims to compare the outcomes of different prophylactic measures on the occurrence of AECOPD after BLVR with EBV.

**Methods:**

We conducted a multicenter, retrospective study of patients who underwent BLVR with EBV at six different institutions. Emphasis was directed towards the specific practices aimed at preventing AECOPD: antibiotics, steroids, antibiotics plus steroids, or no prophylaxis. Subgroups were compared, and odds ratios (ORs) with corresponding 95% confidence intervals (CIs) were calculated.

**Results:**

A total of 170 patients were reviewed. The rate of AECOPD was 21.2% for the full cohort. Patients who received prophylaxis had a significantly lower rate of AECOPD compared with those who did not (16.7% vs. 46.2%; *p* = 0.001). The rate was lowest in patients who received antibiotics alone (9.2%). There was no significant difference in the rate of AECOPD between patients who received steroids alone or antibiotics plus steroids, compared with the other subgroups. The OR for AECOPD was 4.3 (95% CI: 1.8–10.4; *p* = 0.001) for patients not receiving prophylaxis and 3.9 (95% CI: 1.5–10.1; *p* = 0.004) for prophylaxis other than antibiotics alone.

**Conclusions:**

Administration of antibiotics after BLVR with EBV was associated with a lower rate of AECOPD. This was not observed with the use of steroids or in combination with antibiotics.

## 1 INTRODUCTION

Bronchoscopic lung volume reduction (BLVR) has emerged as an important treatment method for patients with severe chronic obstructive pulmonary disease (COPD) compromised by emphysema and air trapping.^1^ Strategies to achieve BLVR include vapor thermal ablation, endobronchial coils, and polymeric lung volume reduction.^2^ Endobronchial valves (EBVs) are currently the only device approved by the Food and Drug Administration to perform BLVR in the United States of America and have been shown to improve quality of life for patients impaired by dyspnea and severe obstructive airway disease.^3^, ^4^

Lung volume reduction decreases static and dynamic lung hyperinflation.^1^ In selected patients, the occlusion of the targeted lung segments promotes passive deflation and limits future aeration, leading to a reduction in lung volumes and air redistribution into less emphysematous regions. The reduction in hyperinflation improves diaphragmatic mechanics, ventilation/perfusion relation, and expiratory airflow, which favors the continuous decrease of lung volumes, regardless of the homogeneity of the emphysema.^1^, ^5^ Most recent literature suggests improved survival in those who achieved complete lobar atelectasis after EBV placement.^2^, ^6^, ^7^

However, the benefits described above can be overcome by adverse events. The main complications after BLVR with EBV are pneumothorax, acute exacerbation of COPD (AECOPD), and, rarely, airway bleeding. The rate of AECOPD varies widely, ranging from 4.6% to 42% in several studies.^2^, ^3^, ^4^, ^5^, ^6^, ^7^, ^8^, ^9^, ^10^ AECOPD increases patients' morbidity, mortality, and healthcare costs and worsens patients' overall quality of life. Reducing its occurrence is imperative to achieve better patient‐centered outcomes and optimize healthcare resources.^11^ Unfortunately, there is little reported on evidence‐based practices to reduce AECOPD after BLVR with EBV.

Physicians must decide to premedicate or not based on their experience and preferences, with no specific guidance. Our study aims to compare the outcomes of different prophylactic measures on the occurrence of AECOPD in patients who underwent BLVR with EBV.

## 2 MATERIALS AND METHODS

We conducted a multicenter, retrospective study of patients who underwent BLVR with EBV from January 2019 to December 2020 in six different institutions, five in the United States and one in Germany. The study was deemed exempt by the institutional review board (#20‐009503). Data regarding clinical and demographic characteristics, pulmonary functions tests, BLVR with EBV procedure, and rate of AECOPD were recorded under an encrypted database. Based on the different approach directed to prevent COPD, patients were divided into two major groups: those who received prophylaxis and those who did not receive prophylaxis. A subgroup analysis was performed according to the types of prophylactic therapy received. Dosage and duration of treatment were also recorded. Across the six institutions, prophylaxis started either the day before or the day of procedure and lasted between 3 and 5 days.

We defined AECOPD as a sustained, acute worsening of the patient's respiratory symptoms beyond normal day‐to‐day variations leading to a change in medication, as stated by the Global Initiative for Chronic Obstructive Lung Disease (GOLD).^12^ Worsening symptoms include an increase in cough level, sputum production, or dyspnea. In this study, we considered an AECOPD event if the criteria above were met within the first 90 days post‐EBV placement.

### 2.1 Statistical analysis

Statistical analysis was performed using IBM SPSS software Version 25.0 (IBM Statistics, Chicago, USA). Continuous data were reported as medians and ranges. Categorical data were reported as frequencies and percentages. Clinical and demographic characteristics between the two major groups were compared using chi‐squared and Fisher's exact tests. Continuous variables were compared using Student's *t* test. We performed logistic regression to ascertain the effect of independent variables on the likelihood of the outcome. Odds ratio (OR) and corresponding 95% confidence intervals (CI) were calculated. All reported *p* values were two sided, and statistically significant difference was considered at *p* < 0.05.

## 3 RESULTS

### 3.1 Patient's baseline characteristics and comorbidities

A total of 170 patients were reviewed. No patient was lost to follow‐up. Baseline characteristics of the full cohort are described in Table 1. The median age, height, and weight were 68.5 years (range: 46–88), 1.65 m (range: 1.47–1.91), and 66.4 kg (range: 33.5–110.0), respectively, and 43% were males. Pulmonary function tests reported a median RV% of 226% (range: 120–504), a median FEV1% of 28% (range: 2.1–86), and a median DLCO% of 30% (range: 11.9–70). The median distance for the 6‐min walk test was 296 m (range: 60–520).

**TABLE 1:** Baseline characteristics based on use of prophylaxis

Baseline characteristics	Prophylaxis (*N* = 144)	No prophylaxis (*N* = 26)	Total	*p* value
Age, median (range)	69.0 (46.0–88.0)	67.5 (46.0–84.0)	68.5 (46.0–88.0)	0.52
Gender				0.17
Female	79 (54.9%)	18 (69.2%)	97 (57.1%)	
Male	65 (45.1%)	8 (30.8%)	73 (42.9%)	
Height (m), median (range)	1.65 (1.47–1.91)	1.64 (1.51–1.80)	1.65 (1.47–1.91)	0.91
Weight (m), median (range)	66.6 (33.6–110.0)	66.4 (47.2–99.0)	66.4 (33.6–110.0)	0.83
Hypertension				0.41
No	79 (54.9%)	12 (46.2%)	91 (53.5%)	
Yes	65 (45.1%)	14 (53.8%)	79 (46.5%)	
Diabetes				1.00
No	128 (88.9%)	24 (9.23%)	152 (89.4%)	
Yes	16 (11.1%)	2 (7.7%)	18 (10.6%)	
Pulmonary hypertension				0.12
No	135 (93.8%)	22 (94.6%)	157 (92.4%)	
Yes	9 (6.2%)	4 (15.4%)	13 (7.6%)	
Chronic kidney disease				0.65
No	136 (94.4%)	24 (92.3%)	160 (89.4%)	
Yes	8 (5.6%)	2 (7.7%)	10 (5.9%)	
Congestive heart failure				0.17
No	141 (97.9%)	24 (92.3%)	165 (97.1%)	
Yes	3 (2.1%)	2 (7.7%)	5 (2.9%)	
RV (%), median (range)	228 (120–504)	219 (155–322)	226.5 (120–504)	1.00
FEV1 (%), median (range)	28.0 (12.0–70.0)	26.0 (12–86.0)	28.0 (12–86.0)	0.42
DLCO (%), median (range)	30.0 (11.9–70.0)	28.0 (19.0–49.0)	30.0 (11.9–70.0)	0.95
6 MWT (m), median (range)	291.5 (72.6–520.0)	318.0 (60.0–477.0)	296.0 (60.0–520.0)	0.27
Number of valves, median (range)	3 (1–9)	4 (2–6)	3 (1–9)	0.006

Abbreviations: 6 MWT, 6‐min walk test; DLCO, diffusing capacity for carbon monoxide; FEV1, forced expiratory volume in 1 s; RV, residual volume.

Patients were divided into two major groups according to whether they received prophylaxis: 144 patients in the prophylaxis group and 26 patients in the no‐prophylaxis group. We found no statistical difference in the clinicodemographic characteristics between the two groups, except for the median number of valves placed (4 vs. 3, *p* < 0.006).

### 3.2 Acute exacerbation of COPD

The rate of AECOPD was 21.2% for the full cohort. Patients in the prophylaxis group had a significantly lower rate of AECOPD compared with the no‐prophylaxis group (16.7% vs. 46.2%; *p* = 0.001) shown in Table 2. In the subgroup analysis, patients were stratified according to the type of therapy received: (1) antibiotics, (2) steroids, (3) antibiotics plus steroids, and (4) no prophylaxis. From the 170 patients, 65 (38.2%) received antibiotics alone, 42 (24.7%) received antibiotics plus steroids, 37 (21.7%) received steroids alone, and 26 (15.3%) received no prophylaxis. Dosage and duration of treatment are described in Table 3.

**TABLE 2:** Acute exacerbation of COPD based on use of prophylaxis

	COPD exacerbation (*N* = 36)	No COPD exacerbation (*N* = 134)	*p* value
Prophylaxis			0.001
No	12 (46.2%)	14 (53.8%)	
Yes	24 (16.7%)	120 (83.8%)	
Antibiotics			0.003
No	30 (28.6%)	75 (71.4%)	
Yes	6 (9.2%)	59 (90.8%)	
Steroids			0.94
No	28 (21.2%)	105 (78.9%)	
Yes	8 (21.6%)	29 (78.4%)	
Antibiotics plus steroids			0.63
No	26 (20.3%)	102 (79.7%)	
Yes	10 (23.8%)	32 (76.2%)	

Abbreviations: COPD, chronic obstructive pulmonary disease.

**TABLE 3:** Dosage and duration of prophylactic medication

Prophylactic medication	Dosage and duration
Antibiotics	
Ampicillin or β‐lactam	250 mg PO q6h × 5 days
Azithromycin	500 mg PO × 1 day + 250 mg PO daily × 4 days or 250 mg PO daily × 5 days
Ceftriaxone	1 g IV daily × 5 days
Levofloxacin	500 mg PO daily × 5 days
Steroids	
Prednisone	40 mg PO daily × 3 days or 40 mg PO daily × 5 days

Abbreviations: IV, intravenous; PO, by mouth.

The rate of AECOPD was significantly lower in the subgroup receiving antibiotics alone compared with the other prophylactic and nonprophylactic approaches (9.2% vs. 28.6%; *p* = 0.003). We found no statistical difference in the rate of AECOPD between the subgroup receiving steroids alone and the other subgroups (21.6% vs. 21.1%; *p* = 0.94), nor between the subgroup receiving antibiotics plus steroids and the other subgroups (23.8% vs. 20.3%; *p* = 0.63), as described in Table 2.

Logistic regression analysis was performed to ascertain the effect of the associated prophylactic measures on the likelihood of COPD exacerbation. Based in the univariable analysis, the OR for AECOPD is 4.3 (95% CI: 1.8–10.4; *p* = 0.001) when no prophylaxis is implemented. For a prophylaxis approach excluding antibiotics alone, the OR for AECOPD is 3.9 (95% CI: 1.5–10.1; *p* = 0.004). When applying the multivariable model, the OR for AECOPD is 2.9 (95% CI: 1.1–7.4; *p* = 0.025) for no prophylaxis and 2.9 (95% CI: 1.8–7.8; *p* = 0.035) for a prophylaxis approach other than antibiotics alone.

## 4 DISCUSSION

The overall rate of AECOPD after BLVR with EBV was 21.2%, and we found a significant decrease in risk of exacerbations when prescribing prophylaxis compared with no prophylaxis in our study. This rate of AECOPD is similar to that reported in previous studies, ranging from 4.6% to 42.3%.^2^, ^3^, ^4^, ^5^, ^6^, ^7^, ^8^, ^9^, ^10^ The wide range in rate is likely due to the subjective clinical diagnosis of COPD exacerbations, the varying practices among centers, and the different postoperative time frames considered, with higher rates presenting in broader periods.^10^ But it also derives from the limited literature on the role of prophylactic therapy to reduce the risk of AECOPD after BLVR, prompting physicians to premedicate or not without a clear guide on how to prevent this frequent complication.

Most studies concerning BLVR with EBV did not describe in detail whether any prophylaxis was implemented, and in fact, the study reporting the lowest rate of COPD exacerbations did not provide specifications regarding their preventive approach.^8^, ^13^ A prospective study compared outcomes from BLVR with EBV versus standard of care (SoC) for severe emphysema in 2012.^9^ Patients who underwent BLVR were treated with a second‐ or third‐generation cephalosporin for 24 h, followed by 7 days of oral therapy. The rate of AECOPD in this group was 11.7% at 90 days, compared with 10% in the SoC group. The study found no significant reduction in the rate of exacerbations with the use of antibiotics between the two groups.^9^ Another prospective multicenter study from 2016 evaluated the efficacy and safety of EBV in patients with homogeneous emphysema, compared with the SoC.^5^ Patients who underwent BLVR were prescribed with intravenous antibiotics for 5 to 7 days. The rate of AECOPD was 16.3% and also did not differ from the rate in patients receiving the SoC.^5^ However, neither study compared the use of antibiotics with other prophylactic measure, and their findings were restricted to the management of emphysema rather than preventing postprocedure complications.

Our study aimed to identify specific prophylactic strategies and found four different practices among the six institutions: (1) antibiotics, (2) steroids, (3) antibiotics plus steroids, and (4) no prophylaxis. The use of any prophylactic measure decreased the risk of AECOPD versus no prophylaxis, but more importantly, antibiotics alone provided the most effective prevention compared with the other practices. We hypothesize this may be related to the diminished lung flora that could potentially trigger an exacerbation. In addition, we had speculated that steroids could provide a more visible impact on reducing the risk of AECOPD, but they did not appear to mitigate this adverse outcome. From the data collected, we consider that the use of steroids should be limited to an established exacerbation rather than a prophylactic measure, as most patients were already under treatment with inhaled corticosteroids.

We acknowledge several limitations to our study that may be addressed in future research on this topic. First, the diagnosis of COPD exacerbations is clinical and subjective; therefore, the number of exacerbations at each center could be under or overdiagnosed. The LIBERATE study evaluating EBV for heterogeneous emphysema proposed a relative contraindication to undergo BLVR as two exacerbations a year leading to hospital stay.^3^ Following GOLD staging, patients in Groups C and D would be relatively contraindicated for this treatment. However, it is important to clarify that patients often present with multifactorial causes for dyspnea, for which air trapping may not be the major source. Although Groups C and D are not absolute contraindications, clinicians should reserve BLVR with EBV for patients in any stage whose main driver for dyspnea is air trapping in the setting of severe emphysema, otherwise benefits will be restricted, and further complications may arise. Second, antibiotic regimens varied among participant institutions, and although this proved to be the most effective preventive therapy, the ideal antibiotic regimen remains unknown. Because a vast number of patients undergo bronchoalveolar lavage with microbiological cultures at the time of valve implantation, this could potentially tailor the antibiotic regimen and distinguish treatment from prevention. Finally, the study is limited by its retrospective design as we were subjected to perform a descriptive analysis of the different practices among bronchoscopists from each institution. The disproportionate number of patients receiving or not prophylaxis emphasizes the need for evidence‐based guidelines that could translate into better patient‐centered outcomes.

Our findings reveal a tendency to prescribe prophylaxis, currently performed without a clear guidance, and set a precedent for future research on preventing AECOPD after BLVR with EBV.

## 5 CONCLUSION

Administration of antibiotics alone after BLVR with EBV was associated with a decreased rate of AECOPD. The use of steroids alone or in combination with antibiotics did not provide the same result. We encourage further research to prevent the risk of this adverse outcome.

## ETHICS STATEMENT

The authors acknowledge that this research work is original and has not been previously published nor is currently being considered for publication elsewhere. All sources used are properly disclosed, and all authors who have been involved are mentioned. This study was approved and deemed exempt by the institutional review board (#20‐009503).

## AUTHOR CONTRIBUTIONS

David Abia‐Trujillo, Sebastian Fernandez‐Bussy, Alejandra Yu Lee‐Mateus, Juan C. Garcia‐Saucedo, and Omran Saifi were responsible for the manuscript conception, literature search, data extraction, data analysis, and manuscript redaction. Felix J. F. Herth, John R. Woytanowski, Ihab Alshelli, Sajive Alevas, Juan P. Uribe Becerra, Adnan Majid, Eric S. Edell, Megan M. Dulohery‐Scrodin, Janani S. Reisenauer, Hiren J. Mehta, Michael A. Jantz, and Hawazin K. Abbas contributed significantly to the data collection, manuscript conception, critical analysis, and manuscript correction. All authors approved the final version of this manuscript.

## CONFLICT OF INTERESTS

The authors have no conflicts of interest to declare.

## Data Availability

The data that support the findings of this study are available on request from the corresponding author. The data are not publicly available due to privacy or ethical restrictions.
